# The impact of diagnosis delay on European patients with generalised myasthenia gravis

**DOI:** 10.1002/acn3.52122

**Published:** 2024-08-01

**Authors:** Elena Cortés‐Vicente, Andras J. Borsi, Charlotte Gary, Wim G.J. Noel, Jennifer M.S. Lee, Wisam Karmous, Qiaoyi Zhang, Kavita H. Gandhi, Alberto E. Batista, Jonathan J. DeCourcy, Sophie G. Barlow, Shiva L. Birija, Gregor A. Gibson

**Affiliations:** ^1^ Neuromuscular Diseases Unit, Department of Neurology Hospital de la Santa Creu I Sant Pau Barcelona Spain; ^2^ EMEA Market Access Janssen‐Cilag High Wycombe UK; ^3^ EMEA Market Affairs Janssen‐Cilag Issy‐les‐Moulineaux France; ^4^ EMEA Market Affairs Janssen Pharmaceutica NV Beerse Belgium; ^5^ EMEA Market Access Janssen‐Cilag A/S Birkerød Denmark; ^6^ EMEA Market Access Janssen‐Cilag Issy‐les‐Moulineaux France; ^7^ Global Market Access Janssen Global Services Titusville New Jersey USA; ^8^ Rare Diseases Adelphi Real World Bollington UK; ^9^ Statistics and Data Analytics Adelphi Real World Bollington UK

## Abstract

**Objective:**

The objective was to determine the mean duration of diagnosis delay for patients with myasthenia gravis from five European countries and explore the impact of >1 year diagnosis delay.

**Methods:**

Patients with myasthenia gravis (*N* = 387) from Europe (France/Germany/Italy/Spain/United Kingdom) and their physicians participated in the Adelphi Real World Myasthenia Gravis Disease Specific Programme™. Diagnosis delay (time from symptom onset to diagnosis) was calculated and characteristics described for patients experiencing >1 year and ≤1 year diagnosis delay. Denominators varied according to outcome as missing data were not imputed.

**Results:**

Mean (standard deviation) diagnosis delay was 363.1 (520.9) days, and 27.1% (105 out of 387) of patients experienced diagnosis delay >1 year. Among patients with >1 year and ≤1 year diagnosis delay, respectively, 69.2% (72 out of 104) and 17.4% [45 out of 259] had initially received a different diagnosis (physician‐reported); 40.0% (42 out of 105) and 24.1% (68 out of 282) were Myasthenia Gravis Foundation of America class III at the time of the survey (physician‐reported); 72.4% (76 out of 105) and 61.3% (173 out of 282) had fatigue (subjective physician reporting from a pre‐selected list of symptoms); 30.5% (32 out of 105) and 17.4% (49 out of 282) had anxiety and 21.9% (23 out of 105) and 13.1% (37 out of 282) had depression (both subjective physician reporting from a pre‐selected list, Likert‐style); and mean (standard deviation) MG‐QoL‐15r score was 14.4 (5.50) and 12.6 (7.84) (self‐reported by *N* = 43 and *N* = 74 patients, respectively).

**Interpretation:**

More than a quarter of patients with myasthenia gravis experienced diagnosis delay of >1 year. These patients had a different clinical profile with regards to severity, symptoms, comorbidities and MG‐QoL‐15r score, compared with patients experiencing ≤1 year diagnosis delay.

## Introduction

Myasthenia gravis (MG) is a rare, chronic, autoimmune disorder of the post‐synaptic membrane at the neuromuscular junction, leading to muscle weakness and fatigability.^1^ Around 85% of patients progress from purely ocular to generalised MG, affecting facial, oral, bulbar, limb and/or respiratory muscles.^2^

Diagnosis of generalised MG can be challenging as muscle weakness, fatigue and other symptoms may be erroneously ascribed to other disorders, including Lambert–Eaton myasthenic syndrome, acute inflammatory demyelinating polyradiculoneuropathy, post‐infection conditions, fatigue syndromes with major psychiatric or social aspects or stroke.^1^, ^3^, ^4^ The fluctuating nature of these symptoms and the presence of comorbidities also complicate diagnosis.^1^, ^3^, ^5^ Furthermore, bulbar symptoms may be associated with a non‐neurological disease such as a digestive or ear, nose and throat condition. Diagnosis may also be hindered by implicit bias, whereby symptoms are less likely to be acknowledged in a particular demographic group. These factors can contribute to a prolonged period of time between symptom onset and accurate diagnosis, referred to as diagnosis delay.

This complexity and the challenges presented to diagnosing clinicians are evident in several expert opinion publications about MG^1^, ^3^, ^5^ and numerous published case reports.^6^, ^7^, ^8^, ^9^, ^10^, ^11^, ^12^ Among patients with MG in Poland, more than 60% reported that the process preceding their MG diagnosis was long and complicated.^13^ As with all rare diseases, timely diagnosis should reduce delays in treatment initiation, decrease patient and caregiver stress and reduce healthcare costs.^14^, ^15^, ^16^, ^17^

A detailed understanding of the occurrence of diagnosis delay across diverse populations of people with MG, as well as the impact this may have on their disease burden and lived experience, is important in informing diagnosis and management strategies. Real‐world data provide an opportunity to investigate this. Adelphi Real World (ARW) Disease Specific Programmes (DSP)™, including the MG DSP, are an established methodology for collecting real‐world data on disease burden and treatment approaches.^18^, ^19^, ^20^ For this study, in particular, we were interested in physician‐reported aspects of the MG diagnosis journey and subsequent treatment and management, as well as clinical characteristics including disease severity and symptom burden and health‐related quality of life (HRQoL) measures as reported by both physicians and patients.

The study objective was to determine the mean duration of diagnosis delay for patients with MG from five European countries, overall and for men and women, and to explore its impact by comparing MG DSP survey response data for patients experiencing >1 year or ≤1 year diagnosis delay.

## Methods

### Study design and data source

A complete description of the ARW DSP™ methodology has been previously published and validated.^18^, ^19^, ^20^ The ARW MG DSP was a cross‐sectional survey with elements of retrospective data collection from physicians and their patients with MG across multiple countries between March and September 2020. This secondary analysis was performed using survey results for physicians and their patients with generalised MG in France, Germany, Italy, Spain and the United Kingdom (UK).

Physicians were hospital‐ or office‐based with a primary specialty of neurology, primary care or geriatrics, who had personally treated ≥1 patient with a confirmed diagnosis of MG in the prior 12 months. They were recruited by local fieldwork agents, with appropriate remuneration for their involvement. Physicians first completed an online survey about disease management, and then completed an online patient record form (PRF) chart review about the next 1–10 consecutive consulting patients with MG seen in clinical practice. Patients were then invited to complete a patient self‐completion (PSC) form voluntarily (without remuneration) and away from physician influence, and results were paired to their physician's responses.

This analysis included only patients meeting the following inclusion criteria: (i) physician‐reported data included timing of both symptom onset and MG diagnosis; (ii) patients categorised by their physician at the time of the survey as MG Foundation of America (MGFA) class II (mild, generalised MG), III (moderate, generalised MG) or IV (severe, generalised MG).^21^ Class I (ocular MG) and class V (defined as requiring intubation, with or without mechanical ventilation, except when employed during routine postoperative management) patients were excluded. If patients used a feeding tube without intubation they would be categorised as class IVb.

### Ethical approval

The study was performed in accordance with relevant guidelines; ethics approval was obtained from the Western Institutional Review Board, approving protocol number: AG8768. All participants provided informed consent. All data were aggregated and de‐identified before receipt; no patient or physician could be identified directly. Data collection was undertaken in line with relevant legislation and guidelines including European Pharmaceutical Marketing Research Association guidelines,^22^ the US Health Insurance Portability and Accountability Act 1996^23^ and Health Information Technology for Economic and Clinical Health Act legislation.^24^

### Outcome measures and definitions

Diagnosis delay was defined as the number of days from time of symptom onset to time of MG diagnosis; both dates were reported in the physician‐completed PRF.

Details of the diagnosis journey, clinical parameters and treatment and management history were also obtained from the physician‐completed PRF. Disease severity was defined using MGFA classification. For ‘remission status’, physicians could choose between the following four options: (i) not in remission – that is, no substantial decrease in clinical manifestations, (ii) minimal manifestations – that is, has some weakness detectable on examination, (iii) in pharmacological remission – that is, has been symptom‐free but continues to take treatment for myasthenia gravis or (iv) in complete stable remission – that is, has been symptom‐free and has not received any treatment for myasthenia gravis during this period.^25^ For response iii or iv, physicians were then asked to state how long the patient had been in remission. The occurrence of symptoms and comorbidities was according to the physician's own definition, selected from a pre‐defined list with the option to also name ‘others’. Data reported as ‘different diagnoses prior to MG diagnosis’ were obtained from the physician's response to the question: ‘Prior to the diagnosis of MG, had the patient ever received any misdiagnoses that were later attributed to the patient's MG?’

Patient HRQoL was assessed in two ways: (i) subjective assessment by physicians using a seven‐point Likert scale, and (ii) using patient‐reported outcome measures obtained from the PSC form. These included the MG‐QoL‐15r (higher scores indicate greater disease impact on HRQoL, and total scores range from 0 to 30)^25^ and the EQ‐5D‐5L, consisting of the EQ‐5D descriptive system (utility score) and the EQ visual analogue scale (EQ VAS; 0–100 from ‘the best health you can imagine’ to ‘the worst health you can imagine’).^26^, ^27^

### Data analysis

For all numerical variables, mean and standard deviation (SD) were reported, and for diagnosis delay, median, minimum and maximum values were also reported. For categorical variables, number and percent in each category were reported. Missing data were not imputed, therefore, the base of patients for analysis could vary by outcome of interest.

Average diagnosis delay was calculated overall and compared between patients with different MGFA class at the time of diagnosis (ANOVA) and between male and female patients (t‐test).

Multivariable linear regression was then performed to estimate the association between diagnosis delay and the independent variables of MGFA class at diagnosis (II or III/IV) and patient sex (male or female); covariates were age at symptom onset (continuous variable), presence of a different diagnosis prior to MG diagnosis (yes or no) and type of healthcare professional who diagnosed MG (general practitioner/primary care physician, neurologist or other). Mean diagnosis delay was then separately predicted for each patient subgroup of interest if all other covariates were taken at their mean values; this is referred to as the adjusted mean. The subgroups of interest were (i) male patients and female patients, and (ii) patients who were MGFA class I, class II and class III/IV at diagnosis (distinct from MGFA class at the time of the survey, as utilised for the study inclusion criteria).

Patients were also categorised as >1 year (>365 days) diagnosis delay or ≤1 year (≤365 days) diagnosis delay. This cut‐off value was close to the mean diagnosis delay observed in our study population, and also aligns with an aspirational goal of the International Rare Diseases Research Consortium: ‘*All patients coming to medical attention with a suspected rare disease will be diagnosed within one year if their disorder is known in the medical literature […]’*.^28^

To explore the diagnosis journey for patients with a longer diagnosis delay, parameters relating to prior diagnoses were described for patients with >1 year or ≤1 year diagnosis delay. In addition, to explore disease burden characteristics with diagnosis delay, patient demographics, clinical characteristics, MG treatment and management history and HRQoL assessments were also described for patients with >1 year or ≤1 year diagnosis delay. For HRQoL, physician‐reported data were analysed both overall (regardless of whether the patient had self‐reported) and also for the sub‐population where both physician and patient had provided HRQoL assessments.

To explore the most common symptom of fatigue, occurrence of physician‐reported fatigue and patient‐reported tiredness (counts and percentages) were reported, and the percentage of patients reporting this when their physician did not – and *vice versa* – were calculated. In addition, parameters (including patient demographics, diagnosis journey, clinical characteristics and HRQoL assessments) were described for patients with or without fatigue. This included any patient in the MG DSP with physician‐reported fatigue (i.e. was not limited to patients included in the diagnosis delay analysis).

To explore the reasons for physicians prescribing particular treatments, the most common reasons for treatment choice were described for first, second and third or later lines (Supplementary Appendix 1). This also included any patient in the MG DSP whose physician provided this information (i.e. was not limited to patients included in the diagnosis delay analysis).

As a sensitivity analysis, for selected parameters identified in the above analyses as having potential importance, values were described for patients with diagnosis delay of > or ≤6 months and > or ≤3 months.

Statistical analyses were performed using STATA v17.^29^

## Results

### Patients

Survey responses for 387 patients with generalised MG included physician‐reported timing of symptom onset and MG diagnosis, and were therefore included in the study. For these patients, mean (SD) age was 52.5 (15.7) years, 54.0% (285 out of 554) were female, mean (SD) body weight was 71.0 (12.6) kg and mean (SD) body mass index was 24.8 (3.6) mg/kg^2^ (Table 1).

**Table 1:** Patient characteristics and diagnosis journey history for patients with myasthenia gravis, overall and stratified according to diagnosis delay of >1 or ≤1 year.

Parameter	*n*	All patients	*n*	Diagnosis delay >1 year	*n*	Diagnosis delay ≤1 year
*Demographic characteristics*						
Age at symptom onset, mean (SD), years	387	47.3 (15.5)	105	46.4 (13.3)	282	47.7 (16.3)
Age category at symptom onset, *n* (%)	387		105		282	
≥50 years of age		177 (45.7)		46 (43.8)		131 (46.5)
≥65 years of age		54 (14.0)		9 (8.6)		45 (16.0)
Age at time of survey, mean (SD), years	387	52.5 (15.7)	105	53.6 (13.2)	282	52.1 (16.5)
Female, *n* (%)	387	209 (54.0)	105	58 (55.2)	282	151 (53.5)
Body weight, kg						
Overall	387	71.0 (12.6)	105	72.2 (12.0)	282	70.5 (12.8)
Male	178	79.1 (9.8)	47	80.1 (9.7)	131	78.7 (9.8)
Female	209	64.1 (10.4)	58	65.8 (9.8)	151	63.4 (10.6)
BMI, mean (SD), kg/m^2^	387	24.8 (3.6)	105	25.1 (3.2)	282	24.7 (3.7)
*Diagnosis journey*						
When patient was diagnosed with MG, months prior to survey, mean (SD)	387	50.3 (68.0)	105	53.6 (73.0)	282	49.1 (66.1)
Patient diagnosed with different condition prior to MG diagnosis, *n* (%)	363	117 (32.2)	104	72 (69.2)	259	45 (17.4)
Number of different diagnoses prior to MG diagnosis, *n* (%)	117		72		45	
1		84 (71.8)		44 (61.1)		40 (88.9)
2		28 (23.9)		24 (33.3)		4 (8.9)
3		4 (3.4)		4 (5.6)		0 (0)
Unknown		1 (0.9)		0 (0)		1 (2.2)
Different diagnoses prior to MG diagnosis (most common), *n* (%)	117		72		45	
Chronic fatigue syndrome		38 (32.5)		24 (33.3)		14 (31.1)
Hysteria		11 (9.4)		11 (15.3)		0 (0.0)
Critical neuropathy/myopathy		11 (9.4)		10 (13.9)		1 (2.2)
Multiple sclerosis		8 (6.8)		4 (5.6)		4 (8.9)
ALS		7 (6.0)		4 (5.6)		3 (6.7)
Oculopharyngeal muscular dystrophy		7 (6.0)		7 (9.7)		0 (0.0)
Posterior circulation stroke		7 (6.0)		1 (1.4)		6 (13.3)
MGFA class at time of diagnosis, *n* (%)	387		105		282	
Class I		61 (15.8)		23 (21.9)		38 (13.5)
Class II		172 (44.4)		49 (46.7)		123 (43.6)
Class III		118 (30.5)		29 (27.6)		89 (31.6)
Class IV		33 (8.5)		4 (3.8)		29 (10.3)
Class V		3 (0.8)		0 (0.0)		3 (1.1)

Diagnosis delay was defined as time from symptom onset to diagnosis of MG.

ALS, amyotrophic lateral sclerosis; BMI, body mass index; MG, myasthenia gravis; SD, standard deviation.

### Diagnosis delay

Overall, mean diagnosis delay (i.e. the time between symptom onset and MG diagnosis) was 363.1 days, or ~ 1 year (Table 2), and the median (min, max) was 183.0 (0.0, 5388.0) days. Of the total population, 27.1% (105 out of 387) of patients experienced >1 year diagnosis delay (diagnosis delay mean [SD] 975.0 [676.4] days; median [min, max] 853.0 [366.0, 5388.0] days) while 72.9% (282 out of 387) of patients experienced ≤1 year diagnosis delay (diagnosis delay mean [SD] 135.3 [107.0] days; median [min, max] 120.5 [0.0, 365.0] days).

**Table 2:** Patient‐reported outcomes for patients with myasthenia gravis, overall and stratified according to diagnosis delay of >1 or ≤1 year.

Parameter	*n*	All patients	*n*	Diagnosis delay >1 year	*n*	Diagnosis delay ≤1 year
MG‐QoL‐15r (patient self‐reported), mean (SD)	117	13.3 (7.10)	43	14.4 (5.50)	74	12.6 (7.84)
EQ‐5D‐5L (patient self‐reported), mean (SD)	122	0.7 (0.24)	46	0.7 (0.19)	76	0.7 (0.26)
EQ‐VAS (patient self‐reported), mean (SD)	120	61.8 (19.89)	47	64.2 (16.02)	73	60.2 (21.98)

EQ‐5D‐5L, EuroQoL 5‐dimension, 5‐level descriptive system; EQ‐VAS, EuroQoL visual analogue scale; MG, myasthenia gravis; MG‐QoL‐15r, revised 15‐item MG QoL questionnaire; SD, standard deviation.

Among the patients who received a relatively prompt MG diagnosis within 1 year of symptom onset, 120 out of 397 (31.0%) received a diagnosis within 3 months, and 197 out of 387 (50.9%) received a diagnosis within 6 months.

Bivariate analysis indicated that there was no significant association between diagnosis delay and patient sex (*p* = 0.6917), but there was a significant association between diagnosis delay and MGFA class at diagnosis (*p* = 0.0014) (Table 3).

**Table 3:** Mean diagnosis delay for patients with myasthenia gravis, overall and stratified by MGFA class at diagnosis and sex.

Patient group	*n*	Mean (SD) diagnosis delay	*p*‐value	Median (min, max) diagnosis delay
All patients	387	363.1 (520.9) days		183.0 (0.0, 5388.0) days
MGFA class at diagnosis				
Class I	61	550.7 (843.2) days	*p* = 0.0014	274.0 (0.0, 5388.0) days
Class II	172	379.3 (468.8) days		212.0 (0.0, 2557.0) days
Class III/IV	154	270.7 (370.4) days		122.0 (0.0, 2253.0) days
Sex				
Male	209	353.4 (435.2) days	*p* = 0.6917	183.0 (0.0, 2253.0) days
Female	178	374.5 (607.4) days		153.0 (0.0, 5388.0) days

Diagnosis delay was defined as time from symptom onset to diagnosis of MG.

MG, myasthenia gravis; MGFA, MG Foundation of America; *n*, number of patients; NS, not statistically significant; SD, standard deviation.

Multivariable linear regression indicated that there were no significant associations between diagnosis delay and either patient sex or MGFA class at diagnosis; adjusted mean diagnosis delays for each subgroup are shown in Table 4.

**Table 4:** Adjusted mean diagnosis delay for patients with myasthenia gravis, overall and stratified by MGFA class at diagnosis and sex.

Patient group	Adjusted mean diagnosis delay	*p*‐Value
All patients	371.1 days	
MGFA class at diagnosis		
Class I	488.1 days	Class I vs II: *p* = 0.383 Class I vs III/IV: *p* = 0.097
Class II	393.3 days	
Class III/IV	301.3 days	
Sex		
Male	346.0 days	Male vs female: *p* = 0.400
Female	401.8 days	

Multivariable linear regression was performed to estimate the association between diagnosis delay and the independent variables of MGFA class at diagnosis (II or III/IV) and patient sex (male or female); covariates were age at symptom onset (continuous variable), presence of a different diagnosis prior to MG diagnosis (yes or no), and type of healthcare professional who diagnosed MG (general practitioner/primary care physician, neurologist or other). Mean diagnosis delay was then separately predicted for each patient subgroup of interest if all other covariates were taken at their mean values; this is referred to as the adjusted mean. Diagnosis delay was defined as time from symptom onset to diagnosis of MG.

MG, myasthenia gravis; MGFA, MG Foundation of America; *n*, number of patients; NS, not statistically significant.

### Diagnosis journey

The survey was conducted a mean (SD) of 50.3 (68.0) months – or around 4 years – after patients received diagnosis of MG; 32.2% (117 out of 363) of patients were diagnosed with different condition(s) prior to this diagnosis of MG (Table 1).

Among patients who experienced >1 year and ≤1 year diagnosis delay, respectively, 69.2% (72 out of 104) and 17.4% [45 out of 259] had initially received a different diagnosis (Table 1). Most commonly, this was chronic fatigue syndrome, hysteria or critical neuropathy/myopathy (Table 1).

A descriptive analysis of selected variables using 3‐month and 6‐month cut‐off values found that trends were broadly comparable to those observed using a 1‐year cut‐off value (Supplementary Appendix 2).

### Clinical characteristics

**Figure 1:**
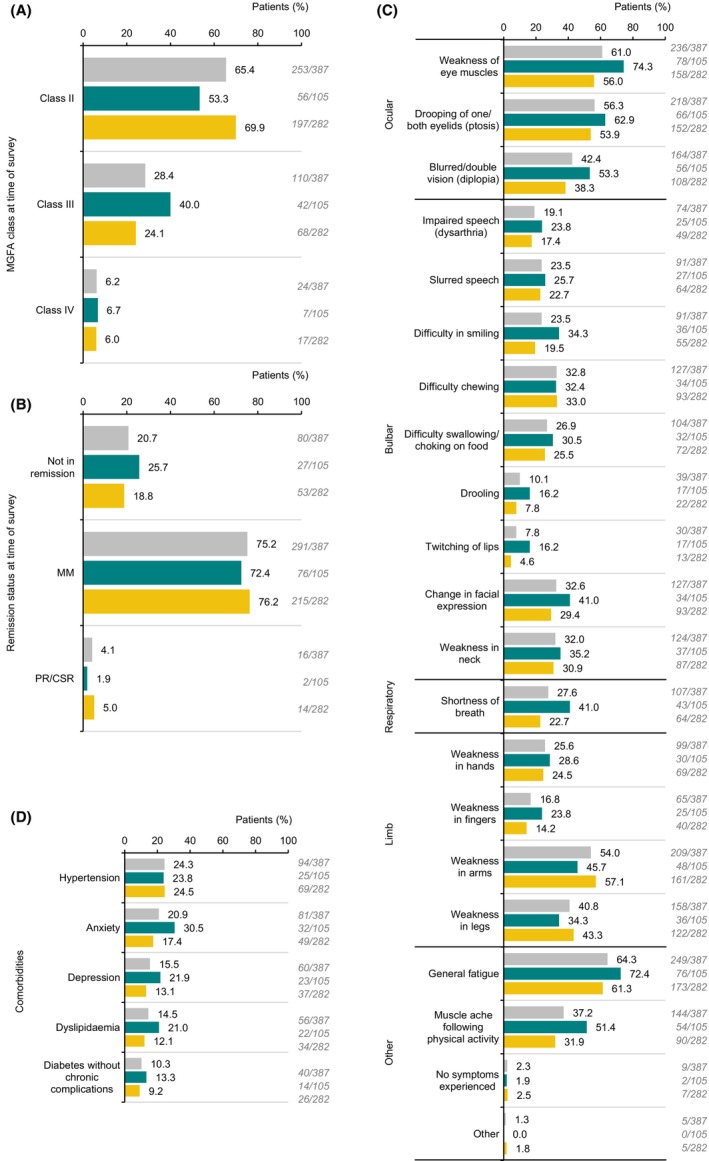
Physician‐reported (A) MGFA class, (B) remission status, (C) presence of symptoms and (D) presence of comorbidities at the time of the survey for patients with MG, overall and stratified according to diagnosis delay of >1 or ≤1 year. CSR, complete stable remission; MG, myasthenia gravis; MGFA, MG Foundation of America; MM, minimal manifestations; PR, pharmacological remission. Key: grey = overall; green = diagnosis delay >1 year; yellow = diagnosis delay ≤1 year.

Overall, physicians reported that the largest proportion of patients (65.4%; 253 out of 387) were MGFA class II (Fig. 1); 20.7% (80 out of 387) of patients were not in remission (i.e. did not have minimal manifestations and were not in pharmacological or complete stable remission; Fig. 1B); 27.2% (102 out of 368) had previously experienced at least one myasthenic crisis (Table 5); symptoms were being experienced at the time of the survey by 97.7% (378 out of 387) of patients (Fig. 1C); and the five most common comorbidities were hypertension, anxiety, depression, dyslipidaemia and diabetes (Fig. 1D).

**Table 5:** Clinical and treatment characteristics, and health‐related quality of life for patients with myasthenia gravis, overall and stratified according to diagnosis delay of >1 or ≤1 year.

Parameter	*n*	All patients	*n*	Diagnosis delay >1 year	*n*	Diagnosis delay ≤1 year
*Clinical and treatment characteristics*						
Patient has undergone thymectomy, *n* (%)	384	92 (24.0)	103	28 (27.2)	281	64 (22.8)
Patient has ever experienced a myasthenic crisis, *n* (%)	368	102 (27.2)	100	31 (31.0)	268	71 (26.5)
Number of myasthenic crises in last 12 months among patients who have experienced a myasthenic crisis, mean (SD)	99	1.0 (0.7)	31	1.1 (0.8)	68	0.9 (0.7)
Severity of fatigue, *n* (%)	249		76		173	
Mild		87 (34.9)		17 (22.4)		70 (40.5)
Moderate		119 (47.8)		38 (50)		81 (46.8)
Severe		43 (17.3)		21 (27.6)		22 (12.7)
Occurrence of symptom ‘tiredness’ (patient self‐reported), *n* (%)	125	98 (78.4)	48	43 (89.6)	77	55 (71.4)
Number of maintenance treatment lines received	387		105		282	
0 lines		11 (2.8)		0 (0)		11 (3.9)
1 line		210 (54.3)		59 (56.2)		151 (53.5)
2 lines		109 (28.2)		33 (31.4)		76 (27)
3 lines		49 (12.7)		13 (12.4)		36 (12.8)
4 or more lines		8 (2.1)		0 (0)		8 (2.8)
Physician‐assessed HRQoL, *n* (%)	387		105		282	
Very poor		2 (0.5)		0 (0)		2 (0.7)
Poor		21 (5.4)		4 (3.8)		17 (6.0)
Somewhat poor		67 (17.3)		24 (22.9)		43 (15.2)
Neither poor nor good		84 (21.7)		25 (23.8)		59 (20.9)
Somewhat good		103 (26.6)		29 (27.6)		74 (26.2)
Good		90 (23.3)		19 (18.1)		71 (25.2)
Very good		20 (5.2)		4 (3.8)		16 (5.7)

Diagnosis delay was defined as time from symptom onset to diagnosis of MG.

HRQoL, health‐related quality of life; MG, myasthenia gravis; SD, standard deviation.

The most common symptom was generalised fatigue (Fig. 1C), reported by physicians for nearly two‐thirds of patients (64.3%; 249 out of 387); this was mild in 34.9% (87 out of 249), moderate in 47.8% (119 out of 249) and severe in 17.3% (43 out of 249) of cases (Table 5). Among 125 patients who completed the patient component of the survey, 78.4% (98 out of 125) reported experiencing tiredness. Concordance between patients and physicians was examined: for 64.8% (81 out of 125), both physician and patient reported fatigue or tiredness; for 13.6%, (17 out of 125) the patient reported tiredness but the physician did not report fatigue; for 4.0% (5 out of 125) the patient did not report tiredness but the physician reported fatigue; and for 17.6% (22 out of 125) neither patient nor physician reported fatigue or tiredness. To further explore fatigue in patients with generalised MG, characteristics were described for all patients in the MG DSP with physician‐reported fatigue (Tables 6 and 7); numerically, these patients were more likely to be female, had a higher mean number of symptoms and were more likely to be in MGFA class III or IV, compared with those without physician‐reported fatigue.

**Table 6:** Patient characteristics, diagnosis journey history, clinical characteristicsand health‐related quality of life measures for patients with myasthenia gravis, overall and stratified according to whether or not patients experienced fatigue.

Parameter	*n*	With physician‐reported fatigue	*n*	Without physician‐reported fatigue
*Demographic characteristics*				
Age, mean (SD), years	362	55.0 (15.59)	192	51.5 (14.87)
Female, *n* (%)	362	199 (55.0)	192	86 (44.8)
BMI, mean (SD), kg/m^2^	362	25.0 (3.59)	192	24.8 (3.69)
*Diagnosis journey*				
When patient was diagnosed with MG, months prior to survey, mean (SD)	331	47.0 (58.0)	163	50.1 (73.2)
Patient diagnosed with different condition prior to MG diagnosis, *n* (%)	306	98 (32.0)	166	36 (21.7)
*Clinical characteristics*				
Severity of fatigue, mild/moderate/severe, *n* (%)	362	114 (31.5)/195 (53.9)/53 (14.6)	192	N/A
MGFA classification, Class II/II/IV, *n* (%)	362	224 (61.9)/112 (30.9)/26 (7.2)	192	140 (72.9)/45 (23.4)/7 (3.6)
Patient has undergone thymectomy, *n* (%)	357	92 (25.8)	184	41 (22.3)
Patient has ever experienced myasthenic crisis, *n* (%)	336	112 (33.3)	169	41 (24.3)
Myasthenic crises in last 12 months, mean (SD)^a^	106	0.9 (0.71)	35	1.1 (0.81)
Physician‐assessed Health‐Related Quality of Life, *n* (%)	357		190	
Very poor		2 (0.6)		0 (0)
Poor		22 (6.2)		6 (3.2)
Somewhat poor		75 (21)		23 (12.1)
Neither poor nor good		85 (23.8)		47 (24.7)
Somewhat good		84 (23.5)		49 (25.8)
Good		79 (22.1)		47 (24.7)
Very good		10 (2.8)		18 (9.5)

BMI, body mass index; HRQoL, health‐related quality of life; MG, myasthenia gravis; SD, standard deviation.

^a^ Among patients who have experienced a myasthenic crisis.

**Table 7:** Patient‐reported outcomes for patients with myasthenia gravis, overall and stratified according to whether or not patients experienced fatigue.

Parameter	*n*	With physician‐reported fatigue	*n*	Without physician‐reported fatigue
MG‐QoL‐15r (patient‐reported), mean (SD)	115	13.9 (6.85)	54	11.8 (6.90)
EQ‐5D‐5L (patient‐reported), mean (SD)	123	0.7 (0.24)	54	0.8 (0.19)
EQ‐VAS (patient‐reported), mean (SD)	122	62.7 (20.38)	52	65.9 (16.01)

EQ‐5D‐5L, EuroQoL 5‐dimension, 5‐level descriptive system; EQ‐VAS, EuroQoL visual analogue scale; MG, myasthenia gravis; MG‐QoL‐15r, revised 15‐item MG QoL questionnaire; SD, standard deviation.

**Figure 2:**
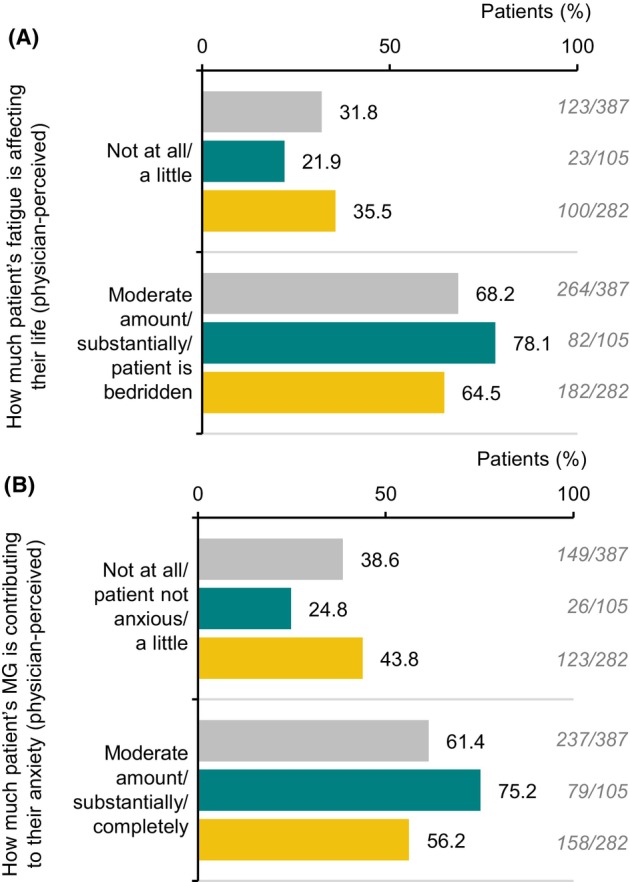
Physician perception about (A) how much patient's fatigue is affecting their life and (B) how much patient's MG is contributing to their anxiety, for patients with MG stratified according to whether they experienced diagnosis delay of >1 year or ≤1 year. MG, myasthenia gravis. Key: grey = overall; green = diagnosis delay >1 year; yellow = diagnosis delay ≤1 year.

Numerical differences were observed between patients with >1 year and ≤1 year diagnosis delay, respectively, for several physician‐reported clinical characteristics (exploratory analysis only). These included the proportion of patients classified as MGFA class II or class III (Fig. 1A); the proportion of patients in remission (Fig. 1B); occurrence of many ocular, bulbar, respiratory, limb and other symptoms except for difficulty chewing and weakness in the arms and legs (Fig. 1C); and occurrence of comorbid anxiety and depression (Fig. 1D). Specifically, among patients with >1 year and ≤1 year diagnosis delay, respectively, physicians reported general fatigue in 72.4% (76 out of 105) and 61.3% (173 out of 282) (Fig. 1C); this was severe in 27.6% (21 out of 76) and 12.7% (22 out of 173) of cases (Table 5), and considered to have a great impact on the patient's life (defined as a moderate amount/substantially/‘patient is bedridden’) for 78.1% (82 out of 105) and 64.5% (182 out of 282) of patients (Fig. 2A). Among patients with >1 year and ≤1 year diagnosis delay, respectively, tiredness was self‐reported by 89.6% (43 out of 48) and 71.4% (55 out of 77) of patients (those who completed the survey only; Table 5). Among patients with >1 year and ≤1 year diagnosis delay, respectively, physicians reported anxiety in 30.5% (32 out of 105) and 17.4% (49 out of 282) (Fig. 1D); they stated that MG contributed to anxiety ‘a moderate amount/substantially/completely’ for 75.2% (79 out of 105) and 56.2% (158 out of 282) of these patients (Fig. 2B); they reported depression in 21.9% (23 out of 105) and 13.1% (37 out of 282) of patients (Fig. 1D).

A descriptive analysis of selected variables using 3‐month and 6‐month cut‐off values found that data and trends were broadly comparable to those observed using a 1‐year cut‐off value. Exceptions were myasthenic crisis (which occurred in 25.6% of those with a ≤3 month delay, vs. 32.2% with a >3 month delay), depression (which occurred at rates of 15–17% regardless of whether patients had > or ≤3 month or > or ≤6 month delay) and fatigue (which occurred in 51.7% of those with a ≤3 month delay, vs. 61.3% with a < 1 year delay) (Supplementary Appendix 2).

### MG treatment and management

**Figure 3:**
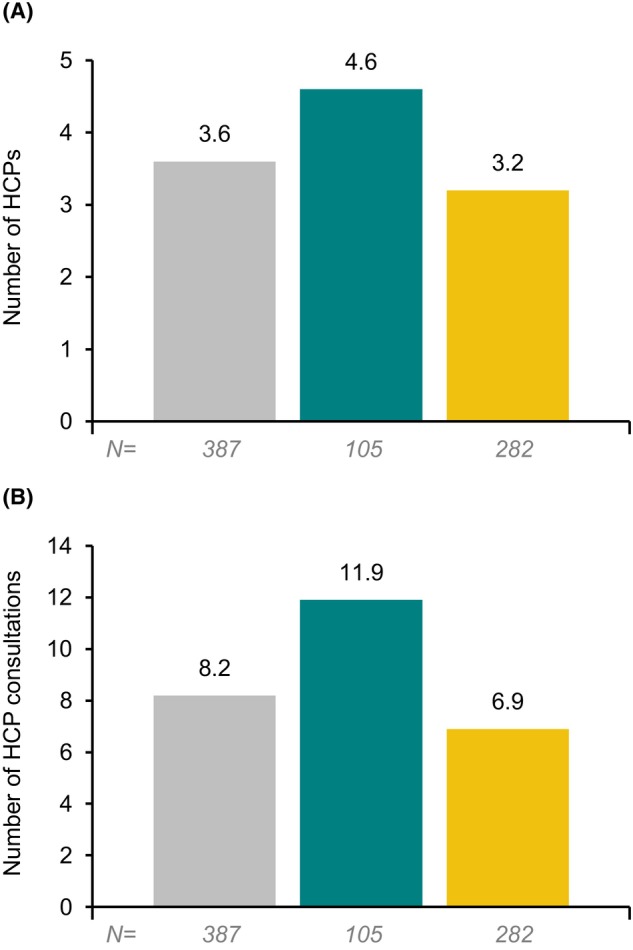
(A) Mean number of HCPs involved in patient management at time of survey and (B) mean number of HCP consultations in 12 months prior to survey, for patients with myasthenia gravis, overall and stratified according to diagnosis delay of >1 or ≤1 year. HCP, healthcare provider; MG, myasthenia gravis. Key: grey = overall; green = diagnosis delay >1 year; yellow = diagnosis delay ≤1 year.

Overall, the vast majority (97.2%; 376 out of 387) of patients had received at least one line of maintenance treatment (Table 5). Among the whole MG DSP population, the most common reasons for treatment choice given by physicians were symptom control at first line (99.6%; 281 out of 282), administration at second line (69.7%; 83 out of 119), and at a third line or later, safety (80.0%; 44 out of 55), suitability (83.6%; 46 out of 55) and general reasons (87.3%; 48 out of 55; this included reasons such as ‘slow down disease progression’, ‘combat a relapse/exacerbation of symptoms’ or ‘maintain quality of life’) (Supplementary Appendix 1). A mean (SD) of 3.6 (1.92) healthcare providers (HCPs) was involved in patient care, with a mean (SD) of 8.2 (6.90) consultations in the 12 months prior to the survey (Fig. 3). Diagnosis was provided by a neurologist in around 80% of patients, and the five most common HCP types involved in patient care were neurologists, general practitioners, pulmonologists, internists and ophthalmologists (Supplementary Appendix 3).

Among patients with >1 year and ≤1 year diagnosis delay, respectively, 100% (105 out of 105) and 96.1% (271 out of 282) of patients had received at least one line of maintenance treatment (Table 5), a mean (SD) of 4.6 (2.17) and 3.2 (1.68) HCPs were involved in patient care (Fig. 2A), and a mean (SD) of 11.9 (8.52) and 6.9 (5.62) HCP consultations had occurred in the 12 months prior to the survey (Fig. 2B). The proportion of patients receiving care from any individual type of HCP was numerically highest for patients with >1 year diagnosis delay, compared with ≤1 year (Supplementary Appendix 3).

### Health‐related quality of life

Overall, HRQoL was assessed by physicians as ‘very poor/poor/somewhat poor’ for 23.2% (90 out of 387), ‘neither poor nor good’ for 21.7% (84 out of 387) and ‘somewhat good/good/very good’ for 55.1% (213 out of 387) of patients (Table 5).

Data were also analysed for a sub‐population of patients who completed the PSC form and their matched physicians. Demographic and clinical characteristics of this group of patients (*N* = 125) were generally comparable with the overall population (*N* = 387) (Supplementary Appendix 4). Physician‐reported HRQoL assessment was ‘very poor/poor/somewhat poor’ for 21.6% (27 out of 125), ‘neither poor nor good’ for 24.0% (30 out of 125) and ‘somewhat good/good/very good’ for 54.4% (68 out of 125) of these patients. Mean patient self‐reported MG‐QoL‐15r score was 13.3 (*N* = 117) and EQ‐5D‐5L score was 0.7 (*N* = 122) (Table 2).

Among patients with >1 year and ≤1 year diagnosis delay, respectively, physician‐reported HRQoL was ‘somewhat poor/poor/very poor’ for 26.7% (28 out of 105) and 22.0% (62 out of 282) of patients overall (Table 5), and 18.8% (9 out of 48) and 23.4% (18 out of 77) for the subgroup of patients who completed the PSC form. In this subgroup, among patients with >1 year and ≤1 year diagnosis delay, mean (SD) MG‐QoL‐15r score was 14.4 (5.50) and 12.6 (7.84), mean (SD) EQ‐5D‐5L score was 0.7 (0.19) and 0.7 (0.24), and mean (SD) EQ‐VAS was 64.2 (16.02) and 63.5 (20.29) (see Table 2 for patient numbers).

A descriptive analysis of selected variables using 3‐month and 6‐month cut‐off values found that trends were broadly comparable to those observed using a 1‐year cut‐off value (Supplementary Appendix 2).

## Discussion

In this study of 387 patients with generalised MG in France, Germany, Italy, Spain and the UK, 27% of patients experienced a diagnosis delay of >1 year between symptom onset and MG diagnosis. The longest delay observed in any patient was over 14 years. Diagnosis delay did not differ significantly between male and female patients. There was a significant association between diagnosis delay and MGFA class at diagnosis, with the longest mean delay of 550.7 days observed in patients initially presenting as MGFA class I (ocular MG), though this difference was not significant in a multivariable analysis. Future studies may examine the differences between patients with different MGFA class at diagnosis, considering both intrinsic patient clinical and demographic characteristics and extrinsic factors such as the specialty of diagnosing physician.

Exploration of survey data indicates that patients with >1 year diagnosis delay may subsequently (in this case, an average of just over 4 years post‐diagnosis) experience a more substantial disease burden, as indicated by MGFA class, occurrence of many ocular and generalised symptoms, occurrence of comorbid anxiety and depression, level of HCP involvement in patient care and impact on patient HRQoL (as indicated by MG‐QoL‐15r score), compared with those who received an MG diagnosis within a year of symptom onset. Future studies may further investigate any associations.

These findings underscore the importance of a timely, accurate diagnosis after MG symptom onset. This has been recognised as a major challenge by the International Rare Diseases Research Consortium.^28^ Studies across many rare conditions, some of which included patients with MG, have shown that diagnosis delay can lead to delayed treatment initiation, increased patient and caregiver stress, and increased healthcare costs.^14^, ^15^, ^16^, ^17^ A registry study of patients in Sweden with MG found that diagnosis delay of ≥2 years was associated with higher disease activity, as quantified by MG‐ADL.^30^

Of note, while the diagnosis of rare diseases within a year of symptom onset is the goal, a sensitivity analysis was also conducted in which patients were categorised as having >3 month versus ≤3 month or >6 month versus ≤6 month diagnosis delay. The trends observed were broadly comparable to the primary analysis with a 1‐year cut‐off.

The mean diagnosis delay for patients in this study was ~1 year. The values observed in previous studies have varied, though our findings are broadly aligned. In a study of 357 patients in the United States (utilising MG DSP data with identical methodology, making the two studies appropriate for comparison), the mean delay was 9 months.^31^ Studies in Germany^32^ and Senegal^33^ reported delays of ~2 years. In Sweden, 59% of patients were diagnosed within 1 year of symptoms, 16% in the second year and 14% after 2–5 years.^30^ In Poland, 44% of patients were diagnosed within 1 year of symptoms, 25% in the second year, 27% between 3 and 10 years and 4% after 10 years.^13^

Our study results shed light on the processes and procedures that may take place between symptom onset and accurate diagnosis of MG. Referred to as the ‘diagnostic odyssey’, for many rare diseases this period comprises multiple assessments and consultations, and can cause considerable stress and uncertainty.^34^, ^35^, ^36^ In our study, nearly 70% of patients who experienced >1 year diagnosis delay had initially received a different diagnosis – most commonly chronic fatigue syndrome, hysteria or critical neuropathy/myopathy – with 33.3% and 5.6% receiving two or even three previous diagnoses, respectively. In a US study, the occurrence of prior misdiagnosis was 19.3% (compared with 32.2% overall in our study); the most common diagnoses there were chronic fatigue syndrome, multiple sclerosis, Guillain–Barre syndrome and connective tissue disease.^31^ In particular, this serves to highlight the challenge of diagnosis when patients present with fatigue and tiredness. General fatigue is challenging to quantify and may initially be assumed to be associated with older age and/or comorbidities. A diagnosis of chronic fatigue syndrome is also perhaps unsurprising. However, MG and chronic fatigue syndrome should be distinguishable as only MG patients have fatigable/variable skeletal muscle weakness.

Gender bias, including the existence of stereotyped preconceptions about the health, behaviour, experiences, needs and wishes of men and women,^37^ is known to influence accurate and appropriate diagnosis and/or treatment of many conditions, ranging from pain,^38^ cardiovascular disease^39^, ^40^, ^41^ and diabetes,^42^ to psychological disorders.^43^ In general, the scientific literature suggests that gender bias may influence the diagnosis received.^44^, ^45^, ^46^, ^47^, ^48^, ^49^ Although there has been limited research into gender differences in diagnosis delay among patients with MG, one study found a significantly longer delay for female than male patients, noting that it was more common for physicians to suspect MG immediately and conduct specific tests in males than in females (patient‐reported data).^13^ No such difference was apparent in our study. Future research may include a similar analysis performed with patients in different geographical areas, as well as analysis of other demographic factors in which implicit bias could play a role.

Age at symptom onset appeared to be comparable in patients with >1 year or ≤1 year diagnosis delay. However, diagnosis of MG in older or elderly patients is known to present particular challenges^50^, ^51^, ^52^ and there is evidence for underdiagnosis among patients with older onset.^53^ Therefore, future studies with a larger patient population, including a greater number of individuals >80 years of age, are required to investigate this further.

Survey responses for the overall population of patients included in this study reflect the disease burden experienced by all individuals with MG. Patient demographics were broadly in line with other, population‐based studies: mean age is in or close to the fifth decade of life and the proportion of patients who are female ranges from 51 to 69%.^31^, ^54^, ^55^, ^56^, ^57^, ^58^, ^59^, ^60^ Some clinical characteristics were different to those in other study populations; for example, 40% of patients with MG in a US registry study had undergone thymectomy^60^ (contrasting with the 24% in our study), other studies included patients with ocular MG^31^, ^54^, ^55^, ^56^, ^57^, ^58^, ^59^, ^60^ (whereas our study included generalised MG only) and among patients in the MyRealWorld MG study,^54^ the most common comorbid condition was thyroid problems in 37.5% of patients (this was not among the most common in our study) and 83.7% had received ≥1 routine MG treatment (lower than the 97.2% in our study). Precise parameters investigated and measures used in each of these studies also varied. Notable features of the overall MG population highlighted by our study include the prevalence and impact of fatigue. Fatigue is an important symptom that lowers quality of life of patients with MG, and that has been the focus of a number of recent studies.^61^, ^62^, ^63^, ^64^ In addition, the importance of depression and anxiety is noted. These mental health disorders were the second and third most common comorbid disorders, though they were less common in this population than in many previous studies.^54^, ^65^ They are considered to be a major concern among individuals with MG, and require increased attention.^65^

Among the patients completing an EQ‐5D‐5L questionnaire, there was no variation in the utility score between those with >1 or ≤1 year diagnosis delay. However, the EQ‐5D‐5L may lack adequate sensitivity and has limitations in fully capturing the overall health state of a patient^62^ and fatigue in particular.^66^ There was a small numerical difference between those with >1 or ≤1 year diagnosis delay in MG‐QoL‐15r score and rating out of 100 on the EQ visual analogue scale.

The study has some limitations. The MG DSP is not a truly random sample of physicians or patients, as participation is influenced by willingness of both and frequency of physician consultations, though it should be noted that there were no formal patient selection verification procedures as these would increase bias towards a particular patient demographic. As with all surveys, recall bias may influence physician responses, though data were collected during appointments, when physicians would be expected to have access to medical records, reducing the likelihood of bias. As missing data were not imputed, the base of patients for analysis could vary between variables; these are clearly stated alongside results.

In conclusion, our analysis of patients with generalised MG from five European countries indicated that patients can experience substantial diagnosis delay, with 27% waiting >1 year for an accurate diagnosis. Furthermore, clinical characteristics and disease treatment/management history varied numerically according to whether patients experienced >1 year or ≤1 year diagnosis delay. These findings underscore the importance of a timely, accurate diagnosis to limit the burden on the patient and healthcare providers, and highlight a need for better disease management strategies. Barriers to diagnosis may potentially be overcome with educational initiatives.

## Funding Information

Janssen‐Cilag EMEA did not influence the original survey through either contribution to the design of questionnaires or data collection. The analysis described here used data from the Adelphi Real World MG DSP. The DSP is a wholly owned Adelphi Real World product. Janssen‐Cilag EMEA is one of multiple subscribers to the DSP. Publication of survey results was not contingent on the subscriber's approval or censorship of the manuscript.

## Conflict of Interest

EC‐V receives public speaking honoraria and compensation for advisory boards and/or consultations fees from Janssen, UCB, Argenx and Alexion. AB, CG, WN, JL and WK are employees of and shareholders in Janssen‐Cilag EMEA or Janssen‐Cilag GCSO. AEB, QZ and KG are employees of Janssen Research & Development US, part of Johnson and Johnson group of companies, and hold stock/stock options of Johnson & Johnson. JdC, SB, SLB and GG are employees of Adelphi Real World.

## Author Contributions

Andras Borsi was responsible for clinical oversight and guidance as lead author. All authors were involved in (1) conception or design, or analysis and interpretation of data; (2) drafting and revising the article; (3) providing intellectual content of critical importance to the work described; and (4) final approval of the version to be published, and therefore meet the criteria for authorship in accordance with the International Committee of Medical Journal Editors guidelines.^67^ In addition, all named authors take responsibility for the integrity of the work as a whole and have given their approval for this version to be published.

## Supporting information


Data S1.

## Data Availability

All data, i.e. methodology, materials, data and data analysis, that support the findings of this survey are the intellectual property of Adelphi Real World. All requests for access should be addressed directly to Gregor Gibson at gregor.gibson@adelphigroup.com.Gregor Gibson is an employee of Adelphi Real World.
