# Supplementary material for: The impact of diagnosis delay on European patients with generalised myasthenia gravis
Source: Ann Clin Transl Neurol. 2024 Aug 1;11(9):2254–67. doi: 10.1002/acn3.52122 (PMC11537147; doi:10.1002/acn3.52122)
Supplement: Supplementary file 1 — Data S1. [file ACN3-11-2254-s001.docx]

**Supplementary Appendices**

**Supplementary Appendix 1**. Reasons for treatment choice provided by physicians

**Methods**

In the patient record form (PRF) physicians were asked the following question, with multiple response options grouped into five categories:

Q. For each **maintenance/chronic** treatment the patient has been prescribed for their myasthenia gravis please record all reasons which influenced your choice in selecting the patient’s **maintenance/chronic** treatment

Disease attributes

- Improve mobility/movement
- Reduce swallowing difficulties
- Reduce speaking difficulties
- Reduce anxiety/depression
- Improve eyelid function
- Improve vision
- Reduce respiratory problems
- Reduce dizziness
- Reduce fatigue/improve sleep
- Reduce risk of myasthenic crises
- Reduce risk of relapse
- Reduce immunogenicity
- Reduce pain
- Reduce muscle weakness

Administration attributes

- Convenient administration
- Clear and simple dosage regimen/ease of titration
- Reduce disruption to patient’s life
- Patient can self-administer
- Once daily dosage
- Availability of different formulations
- Less frequent injections

Side effect/safety attributes

- Improve tolerability
- To minimize glucocorticoid steroid usage
- Less/no interaction with other drugs
- Reduced severity of side-effects
- Reduced risk of complications

Patient type attributes

- Suitable for younger patients
- Suitable for older patients
- Suitable for use in Class I patients
- Suitable for use in Class II patients
- Suitable for use in Class III patients
- Suitable for use in Class IV patients
- Suitable for use across all disease stages

General/other attributes

- Slow down disease progression
- To combat a relapse / exacerbation of symptoms
- Maintain quality of life
- Long-term efficacy
- Fast onset of action
- Good patient compliance
- Cost effective treatment
- Improve work productivity
- Reduce number of hospitalizations
- Reduce duration of hospitalization
- Treatment is reimbursed
- Treatment is covered by insurance
- Treatment is affordable for patient and their co-payer

To explore reasons for maintenance/chronic treatment, responses were described for each treatment line. This included any patient in the Myasthenia Gravis Disease Specific Programme™ with a physician response to this question (i.e. was not limited to patients included in the diagnosis delay analysis).

**Results**

144 physicians reported the current and historic reasons for choice of maintenance/chronic treatment for 529 patients; of these, data were reported for N=282, N=119 and N=55 patients at first line, second line, and third or later lines of treatment, respectively.

‘Symptom control’ reasons were most frequently selected at first line (99.6%), and ‘administration’ at second line (69.7%). ‘Safety’ (80.0%), ‘suitability’ (83.6%) and ‘general’ (87.3%) were selected most frequently at a third line or later.

**Supplementary Appendix 2.**

**Table S1.** Selected patient demographic, clinical, treatment/management, and diagnosis journey characteristics for patients with myasthenia gravis, overall and stratified according to diagnosis delay of > or ≤3 months and > or ≤6 months

| Parameter | Diagnosis delay cut-off 3 months | | | |  | Diagnosis delay cut-off 6 months | | | |
| --- | --- | --- | --- | --- | --- | --- | --- | --- | --- |
|  | **n** | **>3 months** | **n** | **≤3 months** |  | **n** | **>6 months** | **n** | **≤6 months** |
| Demographic characteristics | | |  |  |  |  |  |  |  |
| Age at symptom onset, mean (SD), years | 267 | 47.6 (14.8) | 120 | 46.7 (17.0) |  | 190 | 47.1 (14.4) | 197 | 47.5 (16.5) |
| Age category at symptom onset, n (%) | 267 |  | 120 |  |  | 190 |  | 197 |  |
| ≥50 years of age |  | 130 (48.7) |  | 47 (39.2) |  |  | 93 (48.9) |  | 84 (42.6) |
| ≥65 years of age |  | 31 (11.6) |  | 23 (19.2) |  |  | 17 (8.9) |  | 37 (18.8) |
| Age at time of survey, mean (SD), years | 267 | 53.2 (15.1) | 120 | 51.0 (16.9) |  | 190 | 53.4 (14.4) | 197 | 51.6 (16.8) |
| Female, n (%) | 267 | 137 (51.3) | 120 | 72 (60.0) |  | 190 | 103 (54.2) | 197 | 106 (53.8) |
| BMI, mean (SD), kg/m^2^ | 267 | 24.9 (3.5) | 120 | 24.6 (3.9) |  | 190 | 24.8 (3.7) | 197 | 24.8 (3.4) |
| Diagnosis journey |  |  |  |  |  |  |  |  |  |
| When patient was diagnosed with MG, months prior to survey, mean (SD) | 267 | 50.2 (60.2) | 120 | 50.6 (83.1) |  | 190 | 53.9 (65.2) | 197 | 46.9 (70.5) |
| Patient diagnosed with different condition prior to MG diagnosis, n (%) | 252 | 107 (42.5) | 111 | 10 (9.0) |  | 182 | 94 (51.6) | 181 | 23 (12.7) |
| MGFA class at time of diagnosis, n (%) | 267 |  | 120 |  |  | 190 |  | 197 |  |
| Class I |  | 48 (18.0) |  | 13 (10.8) |  |  | 38 (20.0) |  | 23 (11.7) |
| Class II |  | 124 (46.4) |  | 48 (40.0) |  |  | 94 (49.5) |  | 78 (39.6) |
| Class III |  | 75 (28.1) |  | 43 (35.8) |  |  | 50 (26.3) |  | 68 (34.5) |
| Class IV |  | 20 (7.5) |  | 13 (10.8) |  |  | 8 (4.2) |  | 25 (12.7) |
| Class V |  | 0 |  | 3 (2.5) |  |  | 0 |  | 3 (1.5) |
| Clinical and treatment/management characteristics | | | | |  |  |  |  |  |
| Remission status at time of survey, n (%) | 267 |  | 120 |  |  | 190 |  | 197 |  |
| Not in remission |  | 62 (23.2) |  | 18 (15.0) |  |  | 49 (25.8) |  | 31 (15.7) |
| Minimal manifestations |  | 196 (73.4) |  | 95 (79.2) |  |  | 136 (71.6) |  | 155 (78.7) |
| In pharmacological /complete stable remission |  | 9 (3.4) |  | 7 (5.8) |  |  | 5 (2.6) |  | 11 (5.6) |
| Patient has ever experienced a myasthenic crisis, n (%) | 250 | 64 (25.6) | 118 | 38 (32.2) |  | 176 | 51 (29.0) | 192 | 51 (26.6) |
| Occurrence of symptom ‘general fatigue’ (physician-reported), n (%) | 267 | 187 (70.0) | 120 | 62 (51.7) |  | 190 | 130 (68.4) | 197 | 119 (60.4) |
| MGFA class at time of survey | 267 |  | 120 |  |  | 190 |  | 197 |  |
| Class II |  | 161 (60.3) |  | 92 (76.7) |  |  | 118 (62.1) |  | 135 (68.5) |
| Class III |  | 91 (34.1) |  | 19 (15.8) |  |  | 64 (33.7) |  | 46 (23.4) |
| Class IV |  | 15 (5.6) |  | 9 (7.5) |  |  | 8 (4.2) |  | 16 (8.1) |
| Occurrence of comorbidity ‘anxiety’ (physician-reported), n (%) | 267 | 60 (22.5) | 120 | 21 (17.5) |  | 190 | 44 (23.2) | 197 | 37 (18.8) |
| Occurrence of comorbidity ‘depression’ (physician-reported), n (%) | 267 | 40 (15.0) | 120 | 20 (16.7) |  | 190 | 29 (15.3) | 197 | 31 (15.7) |
| Received at least one line of maintenance treatment, n (%) | 267 | 262 (98.1) | 120 | 114 (95.0) |  | 190 | 187 (98.4) | 197 | 189 (95.9) |
| Number of HCPs involved in patient care, mean (SD) | 267 | 3.9 (2.0) | 120 | 2.8 (1.5) |  | 190 | 4.1 (2.1) | 197 | 3.0 (1.6) |
| Quality of Life |  |  |  |  |  |  |  |  |  |
| Physician-assessed HRQoL, n (%) | 267 |  | 120 |  |  | 190 |  | 197 |  |
| Very poor |  | 1 (0.4) |  | 1 (0.8) |  |  | 0 |  | 2 (1.0) |
| Poor |  | 14 (5.2) |  | 7 (5.8) |  |  | 9 (4.7) |  | 12 (6.1) |
| Somewhat poor |  | 49 (18.4) |  | 18 (15.0) |  |  | 38 (20.0) |  | 29 (14.7) |
| Neither poor nor good |  | 62 (23.2) |  | 22 (18.3) |  |  | 43 (22.6) |  | 41 (20.8) |
| Somewhat good |  | 74 (27.7) |  | 29 (24.2) |  |  | 52 (27.4) |  | 51 (25.9) |
| Good |  | 54 (20.2) |  | 36 (30.0) |  |  | 39 (20.5) |  | 51 (25.9) |
| Very Good |  | 13 (4.9) |  | 7 (5.8) |  |  | 9 (4.7) |  | 11 (5.6) |
| MG-QoL-15r (patient self-reported), mean (SD) | 85 | 14.2 (5.91) | 32 | 10.8 (9.25) |  | 71 | 14.1 (5.85) | 46 | 12.0 (8.6) |

Diagnosis delay was defined as time from symptom onset to diagnosis of MG

ALS, Amyotrophic Lateral Sclerosis; BMI, body mass index; MG, myasthenia gravis; SD, standard deviation

**Supplementary Appendix 3.** Physicians involved in patient management

**Methods**

In the PRF, physicians were asked the following two questions:

Q1. Which physician provided the diagnosis of myasthenia gravis?

Multiple response options:

- Yourself*
- General Practitioner/Primary Care Physician
- Cardiologist
- Dermatologist
- Gastrologist
- Genetic counselor
- Internist
- Metabolic specialist
- Nephrologist
- Neurologist
- Ophthalmologist
- Otolaryngologist
- Paediatrician
- Pain specialist
- Psychiatrist
- Pulmonologist
- Rheumatologist
- Other HCP (specify)
- Don’t know

Q2. Which healthcare professionals have been involved in the overall management of this patient’s myasthenia gravis?

Multiple response options:

- Yourself*
- General Practitioner/Primary Care Physician
- Cardiologist
- Dietician
- Dermatologist
- Gastroenterologist
- Genetic counsellor
- Internist
- Metabolic specialist
- Nephrologist
- Neurologist
- Neuromuscular specialist nurse
- Obstetrician/Gynaecologist
- Ophthalmologist
- Otolaryngologist
- Paediatrician
- Pain specialist
- Psychiatrist
- Pulmonologist
- Rheumatologist
- Other (specify)

**If category ‘yourself’ was selected, this was then recoded to match the specialty of the physician responding.*

To explore differences in disease management, responses were described overall and for patients with >1 year or ≤1 year diagnosis delay

**Results**

Physicians provided information on healthcare professionals for 387 patients (Tables S1 and S2).

**Table S2.** Healthcare professionals involved in diagnosis of myasthenia gravis for patients overall and according to diagnosis delay of >1 or ≤1 year

| **Healthcare professional type, n (%)** | **Overall (N=387)** | **Diagnosis delay >1 year (N=105)** | **Diagnosis delay ≤1 year (N=282)** |
| --- | --- | --- | --- |
| Neurologist | 320 (82.7) | 84 (80.0) | 236 (83.7) |
| General Practitioner/Primary Care Practitioner | 31 (8.0) | 9 (8.6) | 22 (7.8) |
| All other HCP types | 36 (9.3) | 12 (11.4) | 24 (8.5) |

**Table S3.** Healthcare professionals involved in patient management for patients overall and according to diagnosis delay of >1 or ≤1 year

| **Healthcare professional type, n (%)** | **Overall (N=387)** | **Diagnosis delay >1 year (N=105)** | **Diagnosis delay ≤1 year (N=282)** |
| --- | --- | --- | --- |
| Neurologist | 360 (93.0) | 100 (95.2) | 260 (92.2) |
| General Practitioner/Primary Care Practitioner | 314 (81.1) | 89 (84.8) | 225 (79.8) |
| Pulmonologist | 94 (24.3) | 34 (32.4) | 60 (21.3) |
| Internist | 82 (21.2) | 32 (30.5) | 50 (17.7) |
| Ophthalmologist | 81 (20.9) | 30 (28.6) | 51 (18.1) |
| Neuromuscular specialist nurse | 48 (12.4) | 19 (18.1) | 29 (10.3) |
| Psychiatrist | 40 (10.3) | 21 (20.0) | 19 (6.7) |
| Genetic counsellor | 39 (10.1) | 24 (22.9) | 15 (5.3) |
| Metabolic specialist | 26 (6.7) | 17 (16.2) | 9 (3.2) |
| Otolaryngologist | 24 (6.2) | 9 (8.6) | 15 (5.3) |
| Pain specialist | 23 (5.9) | 12 (11.4) | 11 (3.9) |
| Rheumatologist | 18 (4.7) | 11 (10.5) | 7 (2.5) |
| Gastroenterologist | 17 (4.4) | 11 (10.5) | 6 (2.1) |
| Nephrologist | 16 (4.1) | 9 (8.6) | 7 (2.5) |
| Cardiologist | 13 (3.4) | 9 (8.6) | 4 (1.4) |
| Dietician | 11 (2.8) | 5 (4.8) | 6 (2.1) |
| Geriatrician | 10 (2.6) | 4 (3.8) | 6 (2.1) |
| Dermatologist | 5 (1.3) | 3 (2.9) | 2 (0.7) |
| Paediatrician | 3 (0.8) | 2 (1.9) | 1 (0.4) |
| Obstetrician/Gynecologist | 2 (0.5) | 0 (0.0) | 2 (0.7) |
| Other | 22 (5.7) | 0 (0.0) | 22 (7.8) |

**Supplementary Appendix 4.**

**Table S4.** Characteristics of patients overall (data provided by physician, with or without associated patient-reported data), and among patient subgroup for whom data were provided by both physician and patient (N=125)

| Parameter | Overall population^1^ (N=387) | Sub-population^2^ (N=125) |
| --- | --- | --- |
| Age, mean (SD), years | 52.5 (15.7) | 52.9 (13.3) |
| Female, n (%) | 209 (54.0) | 67 (53.6) |
| BMI, mean (SD), kg/m^2^ | 24.8 (3.6) | 25.2 (3.9) |
| MGFA class, n (%) II |  |  |
| Class II | 253 (65.4) | 77 (61.6) |
| Class III | 110 (28.4) | 36 (28.8) |
| Class IV | 24 (6.2) | 12 (9.6) |
| Patient has undergone thymectomy, n (%) | 92 (24.0) | 33 (26.4) |
| Patient has ever experienced a myasthenic crisis, n (%) | 102 (27.2) | 43 (37.4) |
| Patient was not in remission at time of survey, n (%) | 80 (20.7) | 28 (22.4) |
| Number of symptoms experienced at time of survey, mean (SD) | 6.4 (3.8) | 7.0 (3.5) |
| Occurrence of physician-reported general fatigue, n (%) | 249 (64.3) | 86 (68.8) |
| Occurrence of physician-reported anxiety, n (%) | 81 (20.9) | 27 (21.6) |
| Occurrence of physician-reported depression, n (%) | 60 (15.5) | 19 (15.2) |
| When patient was diagnosed with MG, months prior to survey, mean (SD) | 50.3 (68.0) | 52.9 (58.0) |

^1^Physician-reported data +/- patient-reported data; ^2^Both physician and patient-reported data, as included in analysis of health-related quality of life data

BMI, body mass index; MG, myasthenia gravis; MGFA, MG Foundation of America; n, number of patients; NS, SD, standard deviation
